# Studying Performance and Kinetic Differences between Various Anode Electrodes in Proton Exchange Membrane Water Electrolysis Cell

**DOI:** 10.3390/ma15207209

**Published:** 2022-10-16

**Authors:** Zhenye Kang, Zihao Fan, Fan Zhang, Zhenyu Zhang, Chao Tian, Weina Wang, Jing Li, Yijun Shen, Xinlong Tian

**Affiliations:** 1 State Key Laboratory of Marine Resource Utilization in South China Sea, Hainan Provincial Key Lab of Fine Chemistry, School of Chemical Engineering and Technology, Hainan University, Haikou 570228, China; 2 Hainan New Energy Investment Co., Ltd., State Power Investment Corporation, Limited (Hainan), Haikou 570100, China

**Keywords:** water electrolysis, hydrogen production, electrode, kinetics, ink composition

## Abstract

The electrode, as one of the most critical components in a proton exchange membrane water electrolysis (PEMWE) cell for hydrogen production, has a significant impact on cell performance. Electrodes that are fabricated via various techniques may exhibit different morphologies or properties, which might change the kinetics and resistances of the PEMWE. In this study, we have successfully fabricated several electrodes by different techniques, and the effects of electrode coating methods (ultrasonic spray, blade coating, and rod coating), hot press, and decal transfer processes are comprehensively investigated. The performance differences between various electrodes are due to kinetic or high frequency resistance changes, while the influences are not significant, with the biggest deviation of about 26 mV at 2.0 A cm^−2^. In addition, the effects of catalyst ink compositions, including ionomer to catalyst ratio (0.1 to 0.3), water to alcohol ratio (1:1 to 3:1), and catalyst weight percentage (10% to 30%), are also studied, and the electrodes’ performance variations are less than 10 mV at 2.0 A cm^−2^. The results show that the PEMWE electrode has superior compatibility and redundancy, which demonstrates the high flexibility of the electrode and its applicability for large-scale manufacturing.

## 1. Introduction

Sustainable energy systems always include various renewable energy sources, such as wind, solar, hydro, etc., but those renewable energy sources are intermittent within hours, days, or even seasons, which are nonreliable and cannot be directly connected to the current electric grid [1,2,3,4]. Therefore, an ideal energy carrier that can mitigate the differences between energy supplies and demands is critical for developing sustainable energy systems [5,6,7,8,9]. Hydrogen accounts for about only 2% of the world energy consumption at present, while it has been proposed as one of the most promising energy carriers in the next few decades by most of the governments in the world to achieve carbon emission reductions [10,11,12,13], due to its high energy density, low weight, environmentally friendly, and abundant reserves in water [14,15,16,17,18]. Proton exchange membrane water electrolysis (PEMWE) has been regarded as a promising technology for hydrogen production via water splitting [19,20,21,22]. PEMWE can be operated at a high current density that enables high hydrogen production rate [23,24,25,26], and quickly starts or stops, which fits well with intermittent renewable energy sources [27]. Therefore, a lot of researchers have paid attention to PEMWE technology and targeted at its commercialization, due to its advantages compared to other hydrogen production techniques [28,29,30,31,32]. 

**Figure 1:**
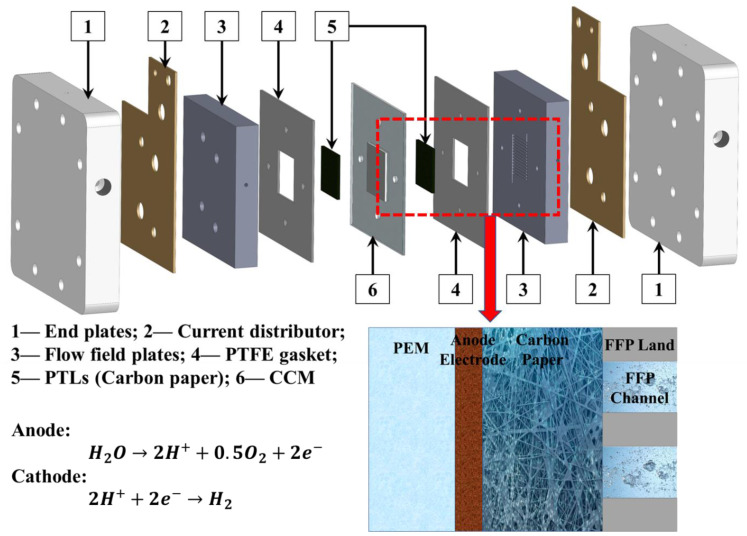
Schematic of typical PEMWE cells and components around anode electrode.

In a PEMWE device, one of the main components is the membrane electrode assembly (MEA), which typically consists of catalyst-coated membrane (CCM) and porous transport layers (PTLs) [33], as shown in Figure 1. In a CCM, there is a piece of Nafion membrane that is sandwiched by two catalyst layer (CL) electrodes on each side (anode and cathode). MEA directly determines the overall PEMWE performances, including the electrochemical reaction kinetics, cell ohmic losses, and diffusion losses [34]. Usually, noble metals are utilized as catalyst materials, for example, iridium/ruthenium-based materials for anode oxygen evolution reaction (OER) and platinum-based materials for cathode hydrogen evolution reaction (HER) [35,36,37]. Due to the high cost of the noble metal catalysts, many researchers have focused on developing electrochemical catalysts with low noble metal loadings or non-noble metals [38,39,40]. Most of the catalysts were only characterized in a three-electrode cell or half-cell [41], which lacks the device performance, and their real performance in PEMWE devices is still a mystery. When applying catalyst materials into a PEMWE device, the first step is to fabricate the electrode and optimize the electrode fabrication method and its structure, which might affect the kinetics of the catalyst. Therefore, understanding the effects of electrode fabrication method is a prerequisite for new catalyst characterization in a PEMWE device. 

Currently, the most widely used techniques for electrode fabrication include ultrasonic spray, doctor blade coating, rod coating, slot-die coating, painting or brushing, and other novel methods. However, only a few researchers have studied the effects of electrode fabrication methods. For example, Zhang et al. have investigated the electrodes fabricated via decal transfer and direct deposition for CCMs, and they found that CCMs fabricated with a direct spray deposition method displayed better cell performance compared to CCMs fabricated with a decal transfer method, which is mainly due to greatly reduced ohmic resistances and improved mass transport [42]. They also developed an advanced patterned electrode that can achieve good performance and found that the catalyst mass activity can be significantly improved [43]. However, the kinetics of the electrodes were not analyzed in their works. Gao et al. developed a novel electrode structure, which contains an ultra-thin gold layer within the electrode. The novel electrode can enhance the electron transport and improve the catalyst utilization, which has been demonstrated as an effective way for improving the electrode kinetics and performance [44]. Liu et al. studied the recent progresses of MEA, and they found that the distribution and thickness of ionomers in the CL should be modulated to further improve ion conduction, mass transport, and catalyst utilization [45]. Park et al. successfully fabricated the MEA by using roll-to-roll method, which showed similar performance compared to ultrasonic sprayed MEAs, and thus they demonstrated a promising direction for large-scale and high-efficient production of MEA for PEMWE [46]. Although there have been several studies focused on investigation of electrode fabrication, a comprehensive and systematic analysis of electrode fabrication is still lacking.

It also should be mentioned that when fabricating an electrode, most of the techniques require preparation of catalyst ink, and then a deposition process with or without pre/post-treatment. Thus, the composition of the catalyst ink has a great impact on the electrode morphologies and properties, which may result in different performance. Commonly, catalyst ink is composed of solid catalysts, Nafion ionomer dispersion, and solvent. Nafion ionomer in the dispersion is used as a bifunctional material, which is for conducting protons and binding metal catalyst particles within electrodes. Currently, the most widely used solvent includes water, alcohol, or other surfactants. Therefore, catalyst ink is a kind of complex suspension, and the compositions might have some effects on electrode properties and performance. The effects of catalyst ink compositions on MEA performance in PEMWE devices have not been comprehensively investigated and reported yet. 

In this work, we have fabricated CCMs by using several typical techniques, including ultrasonic spray, blade coating and rod coating, and investigated the effects of decal transfer and hot press processes. In addition, the catalyst ink with different water to alcohol ratios, ionomer contents, and solid metal catalyst contents were studied comprehensively. Various CCMs were characterized in a PEMWE cell, and the polarization curve and electrochemical impedance spectroscopy (EIS) were measured to analyze the performance and kinetic differences. This work provides a comprehensive understanding of the electrode fabrication methods and ink compositions on PEMWE cell performance and helps the large-scale production of MEA for hydrogen production systems.

## 2. Experimental Details

### 2.1. Materials

Pt on carbon (Pt/C) powder from Tanaka Kikinzoku Kogyo TEC10E50E with 50 wt% Pt was used as the cathode catalyst, and IrO_2_ powder from Macklin with 99.9% purity was used as the anode catalyst. DI water (18.2 MΩ cm) was made from Heal Force Water Purification System. The n-propanol (>99.5% nPA from Macklin) and Chemours D520 Nafion dispersion were used for catalyst ink preparations as received. A piece of Nafion 115 membrane (DuPont) was used as the material for CCM fabrication. Carbon paper TGP-H-060 from Toray with approximately 78% porosity and 190 μm thickness was used as both anode and cathode PTLs for all short-term performance tests [47], and fresh PTLs were always used when the cell was reassembled.

### 2.2. Electrode Fabrication

**Figure 2:**
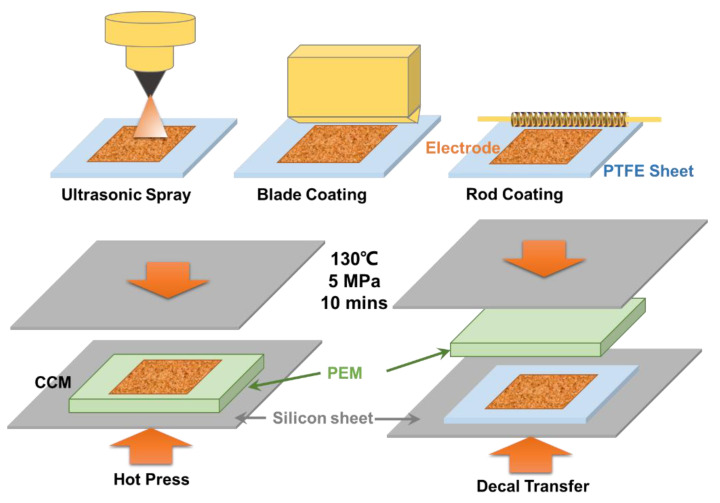
Schematic of the coating technique and electrode fabrication procedures.

Various electrodes were fabricated in this study and the details are shown in Table 1 and Figure 2. Ultrasonic spray was processed by using the UAM4000L ultrasonic spray system from CHEERSONIC. All the CCMs had an ultrasonic sprayed cathode Pt/C electrode, with approximate 0.45 ionomer to carbon ratio and 0.2 mg cm^−2^ Pt loading [48]. For the anode electrodes, the effects of coating techniques and post-treatments were investigated. For the SP, SP + HP, and SP + DT samples, the effects of hot press and decal transfer can be analyzed. The catalyst ink had an ionomer to catalyst weight ratio of 0.2 for the sprayed IrO_2_ anode, with a 2:1 water to nPA volume ratio that was the same for cathode. Then, the effects of coating methods between ultrasonic spray, rod coating and blade coating (as shown in Figure 2) were investigated with SP + DT, RC + DT, and BC + DT samples. For the catalyst ink, the default composition was 0.2 ionomer to catalyst ratio, 2:1 water to nPA ratio, and 20% catalyst weight percentage. For all the catalyst ink preparation, solid catalyst powders were weighted and moved into a glass bottle, then DI water and nPA was added in sequence, and at last the ionomer dispersion was dropped into the mixture by pipette which was washed by the upper clear mixture 2–3 times to make sure the right amount of ionomer dispersion was added due to its high viscosity. The catalyst ink mixture was ultrasonicated in ice water for 30–40 min before the coating. For the rod and blade coating, the PTFE sheet substrate was taped on the coating machine (MSK-AFA-SC200) and maintained at 55 °C for at least 10 min. The coating speed was fixed at 60 mm/s, and the coated samples was moved to an oven for drying at 80 °C for 10 min. After drying, the catalyst-coated PTFE sheet was moved to a hydraulic compression system (NL-600D from Nuoxinda Tech. Corp., Tianjin, China) and the decal transfer process is shown in Figure 2. For the hot press or decal transfer process under the hydraulic compression system, the compression conditions were 5 MPa and 130 °C for 10 min. 

### 2.3. Characterization

The CCMs were tested in a typical home-made PEMWE hardware, which is also shown in Figure 1. The endplates, current distributor, and flow field plates were made of aluminum, gold-coated copper, and platinum-coated titanium, respectively. The cell has an active area of 5 cm^2^ with parallel flow channels. During the test, DI water was pumped to the anode side of the PEMWE with a flow rate of 50 mL min^−1^ and the water temperature was controlled at the inlet of the hardware at 80 °C. The cell temperature was also controlled at 80 °C in the anode flow field plate by using the two adhesive heating pads outside of two endplates. The backpressure of the anode and cathode was ambient, which is 0.98–1.01 bar. 

The cell was tested by using the Gamry instrument (Reference 3000 and 30 K booster). First, the cell was conditioned at 0.1 A cm^−2^, 1.0 A cm^−2^, and 1.7 V for 30 min, 30 min, and 2 h, respectively, after each new CCM was assembled. Then, the performance was measured from low to high current densities in 19 steps, while the EIS was measured below 1.0 A cm^−2^ for the electrode kinetic characterizations. High frequency resistance (HFR) was extracted from the left intercept with the x-axis in the Nyquist plots from EIS data. Three samples of each case were prepared and tested to check the repeatability and obtain the error bars.

## 3. Results and Discussions

### 3.1. Effects of Anode Electrode Fabrication Methods

**Figure 3:**
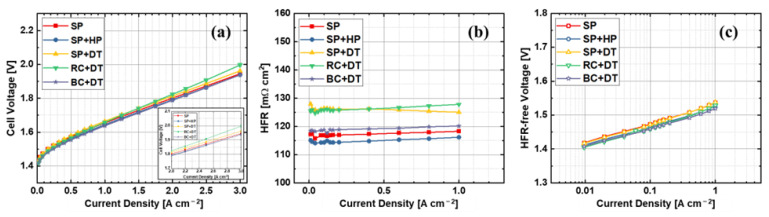
Results comparison between various anode electrode fabrication methods. (**a**) Performance, (**b**) HFR, (**c**) Tafel slopes.

**Figure 4:**
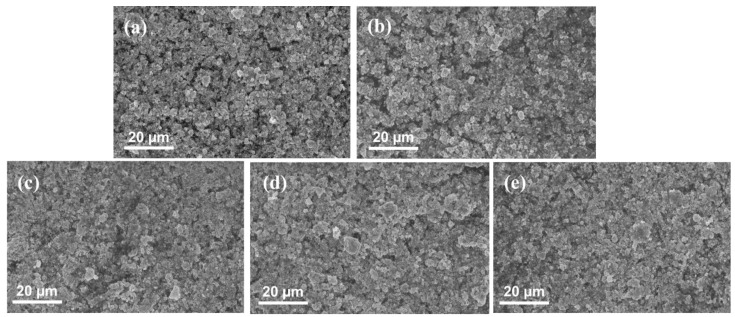
SEM images of various anode electrodes (**a**) SP, (**b**) SP + HP, (**c**) SP + DT, (**d**) RC + DT, (**e**) BC + DT.

Figure 3 shows the results comparison between different anode electrodes that were fabricated by various techniques or procedures. The error bars are similar or even smaller than the symbols of the plots, indicating a good repeatability of the CCMs. The performance between various anode electrodes changes mainly at high current density range, which is mainly due to different HFR values, as shown in Figure 3b. It is found that the hot press process has limited effects on the PEMWE performance, while the decal transfer process results in a higher HFR and slightly worse performance by comparing SP, SP + HP, and SP + DT samples. The hot press process may slightly reduce the membrane thickness due to the compression under high pressure, which leads to a smaller HFR value for the SP + HP sample. However, the decal transfer process will significantly increase the HFR between SP + HP and SP + DT. The results indicate that the decal transfer process may change the interfacial contact between the electrodes and PEM, which results in higher HFR values, and this has been reported before in PEM fuel cells [49]. The kinetics between SP and SP + DT samples are identical, while the SP + HP sample shows slightly smaller voltage, which can be seen from Figure 3c. It is assumed that the high compression (5 MPa at 130 °C for 10 min) hot press process may contribute to better active sites distribution within the CL, but this cannot be observed with SEM images (Figure 4a,b). When applying rod coating technique, a significantly worse performance is obtained, which is also mainly due to the large HFR values. However, when the electrode is fabricated by blade coating, a good performance is achieved and its HFR value is smaller than rod-coated electrodes, which is also comparable to sprayed samples. The HFR variations between rod and blade coating may be caused by the surface flatness of the final electrodes, since the rod coating has a grooved rod, while the blade coating uses a smooth and straight blade for coating (shown in Figure 2). The performance results indicate that electrode fabrication methods have some impacts the PEMWE performance by influencing the HFR or kinetics, but the biggest deviation of performances between different electrodes is less than 30 mV at 2.0 A cm^−2^ (shown in Table 2). The HFR-free voltages shown in Figure 3c and Table 2 indicate that the kinetics between various anode electrodes are similar, and the Tafel slopes have a variation between 56–60 mV dec^−1^. The results indicate that anode electrodes have a high redundancy in PEMWE, which is different compared to PEM fuel cells. The SEM images in Figure 4 show that the catalyst layer morphology is similar between various electrodes that are fabricated by different techniques. It also demonstrates that the anode electrode in PEMWE has a high flexibility, and it is not very sensitive to electrode fabrication methods.

**Figure 5:**
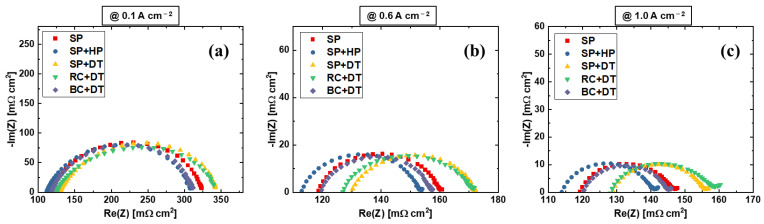
EIS comparison at (**a**) 0.1 A cm^−2^, (**b**) 0.6 A cm^−2^, and (**c**) 1.0 A cm^−2^ for various anode electrodes.

Figure 5 shows the Nyquist plots of the EIS at different operating current densities. The HFR can be extracted from the left intercept of the x-axis, and the distance of the semi-arc indicates the kinetics of the PEMWE, which is mainly rely on the electrode properties [50,51]. The arc size decreases with increasing current density, which follows the Butler-Volmer theory [48]. The kinetics of various electrodes show similar characteristics, which is in agreement with the Tafel results in Figure 3c and Table 2. The results confirm that various anode electrode fabrication methods have negligible impact on the electrode kinetics, while they do impact the ohmic resistances, including the electrode resistances and interfacial contact resistances. The decal transfer process for the sprayed electrodes will result in worse performance, which could probably be the effects of Nafion skin layers that formed and the graded ionomer distribution in the electrode at thickness direction during the multilayer spray process [49]. After a decal transfer process, the Nafion skin layer will be located on top of the CL that is in contact with PTLs, and due to its non-electrical conduction nature, the interfacial contact resistance between PTL and CL increases. However, for the one-path coating technique, the blade coating shows better performance compared to rod coating, which is also better than the SP + DT electrode. The electrode surface flatness may explain the performance differences between rod coating, which uses a grooved rod, and blade coating, which uses a straight blade.

### 3.2. Effects of Anode Electrode Ink Composition

**Figure 6:**
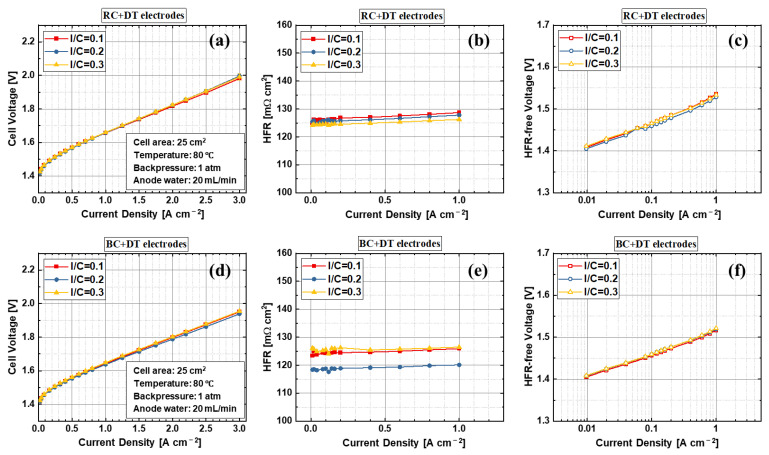
Effects of the ionomer to catalyst ratio in anode electrode ink (**a**,**d**) performance, (**b**,**e**) HFR, and (**c**,**f**) Tafel slopes with two fabrication methods: (**a**–**c**) rod coating and decal transfer, and (**d**–**f**) blade coating and decal transfer.

In the electrode, the ionomer covers the solid catalyst particles’ surface and forms a 3D porous structure, which conducts the protons and helps to bind the catalyst particles together. When the ionomer content is low, both of the two functions may be impacted, resulting in poor kinetic and performance due to significantly reduced proton transport path and therefore decreased active sites. When the ionomer content is high, ionomers may cover all the catalyst particles and fulfill the pores inside the electrode, which will hinder the mass transport of reactant water and product oxygen. Therefore, an appropriate ionomer content is critical for electrode development. Figure 6 shows the results of the rod coating and blade coating electrodes with different ionomer to catalyst ratios during the ink preparation. The results show that the ionomer to catalyst ratio between 0.1 to 0.3 has nearly no impact on PEMWE cell performance of both RC + DT and BC + DT samples. The HFR and kinetics of the samples with various ionomer to catalyst ratios show almost identical results, indicating that the electrodes of the PEMWE have a wide range of ionomer to catalyst ratio compatibility. However, for the RC + DT electrodes, the 0.2 ionomer to catalyst ratio show slightly smaller HFR, but it has no obvious effects on PEMWE cell performance, as shown in Figure 6d. The blade-coated samples also demonstrate a better performance than rod-coated samples due to the electrode surface flatness, which agrees with the previous results. Therefore, the results indicate that the ionomer to catalyst ratio between 0.1 and 0.3 is an ideal range for anode electrode in PEMWE.

**Figure 7:**
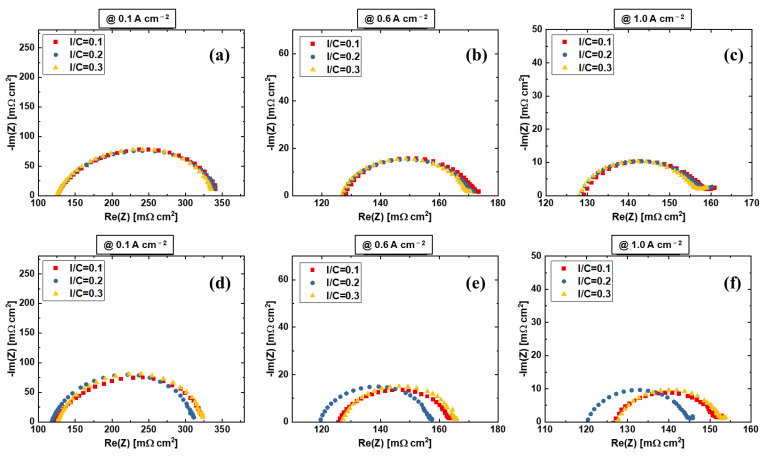
EIS comparison at (**a**,**d**) 0.1 A cm^−2^, (**b**,**e**) 0.6 A cm^−2^, and (**c**,**f**) 1.0 A cm^−2^ with two fabrication methods: (**a**–**c**) rod coating and decal transfer, and (**d**–**f**) blade coating and decal transfer.

Figure 7 shows the EIS results of the rod-coated and blade-coated samples at different current densities. The HFR and kinetics of the rod-coated samples are identical from low to high current densities. It is interesting that a second small semi-arc appears at 1.0 A cm^−2^, which represents transport loss and usually includes two parts that are mass transport of reactants and products, and ion transport within the electrodes and at the interfaces of each component. The blade-coated samples have a smaller HFR for the 0.2 ionomer to catalyst ratio electrode, and the kinetics are identical. At 1.0 A cm^−2^ current density, we do not see a second semi-arc, representing a better transport compared to rod-coated electrodes. We speculate that the ion transport loss at the interfaces between CL and PEM is the main reason for this, since they have identical ink composition and similar electrode morphologies at top view (Figure 4). As mentioned above, rod coating uses a grooved rod while the blade coating uses a smooth blade for the coating, which may result in a different flatness of the electrode surface. This surface will face the PEM after the decal transfer process. Therefore, we assume that the second semi-arc is mainly caused by the ion transport at the interfaces. The results demonstrate that the blade-coated electrodes is better than rod-coated electrodes mainly due to better electrode structure that results in better ion transport at interfaces. However, the kinetics of the electrodes of rod-coated and blade-coated samples are almost the same, showing a good compatibility of the PEMWE electrodes.

**Figure 8:**
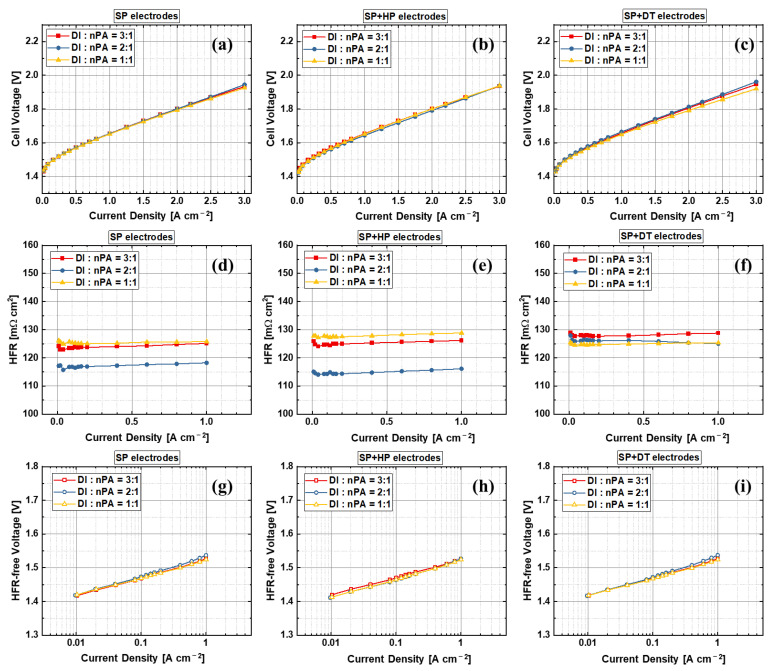
Effects of water to nPA ratio in anode electrode ink (**a**–**c**) performance, (**d**–**f**) HFR, and (**g**–**i**) Tafel slopes with different anode electrode fabrication methods (**a**,**d**,**g**) direct ultrasonic spray, (**b**,**e**,**h**) ultrasonic spray and hot press, and (**c**,**f**,**i**) ultrasonic spray and decal transfer.

Figure 8 shows the effects of the water to alcohol ratio of the sprayed samples during the catalyst ink preparation. For the SP and SP + HP electrodes, the effects of water to nPA on PEMWE cell performance are negligible. However, the HFR of the 2:1 water to nPA ratio is slightly smaller than the 1:1 and 3:1 samples. Their performance is almost identical, and the HFR difference has a limited impact on final PEMWE performances because of its small variations. It is interesting that the water to nPA ratio seems have an impact on PEMWE cell performance for the SP + DT electrodes, while having no impact on HFR. The 1:1 water to nPA ratio SP + DT electrode achieves slightly better performance, which is mainly due to the better kinetics. The Tafel plots in Figure 8i show that the kinetics are slightly varied between different water to nPA ratios, and the trend is the same with the performance. This may be mainly due to the ionomer skin layer formation during the sprayed and decal transfer process, which do not exist for the SP and SP + HP electrodes. With a decal transfer process, the sprayed top-surface will be adhered to the PEM, and the bottom surface on the PTFE sheet will be top-facing to PTLs.

**Figure 9:**
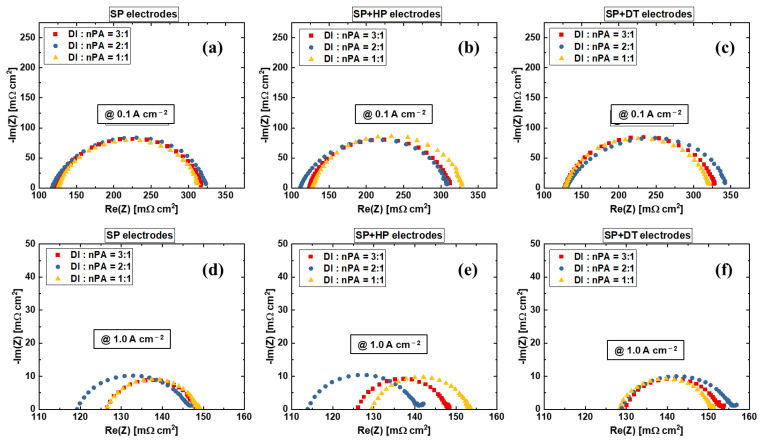
EIS comparison at (**a**–**c**) 0.1 A cm^−2^ and (**d**–**f**) 1.0 A cm^−2^ with different anode electrode fabrication methods (**a**,**d**) direct ultrasonic spray, (**b**,**e**) ultrasonic spray and hot press, and (**c**,**f**) ultrasonic spray and decal transfer.

Figure 9 shows the EIS results of the electrodes with various water to alcohol ratios. The SP + DT electrodes have slightly higher HFR values compared to SP and SP + HP electrodes. The kinetics of the electrodes with 2:1 water to alcohol ratio are slightly larger than the electrodes with 1:1 or 3:1 water to alcohol ratios, which is almost unobservable in Tafel plots (Figure 8g–i)), and this impact is so limited to reflect on cell performances. For the EIS at 1.0 A cm^−2^ shown in Figure 9f, it is clear that the kinetics of the 1:1 water to nPA ratio are smaller than 2:1 and 3:1 SP + DT electrodes. The results indicate that the decal transfer process does have an impact on PEMWE electrode performance, which is in agreement with the results in Figure 3 and Figure 5. In general, the effects of the water to nPA ratio are limited and the anode electrode in PEMWE also shows a high compatibility of this parameter.

**Figure 10:**
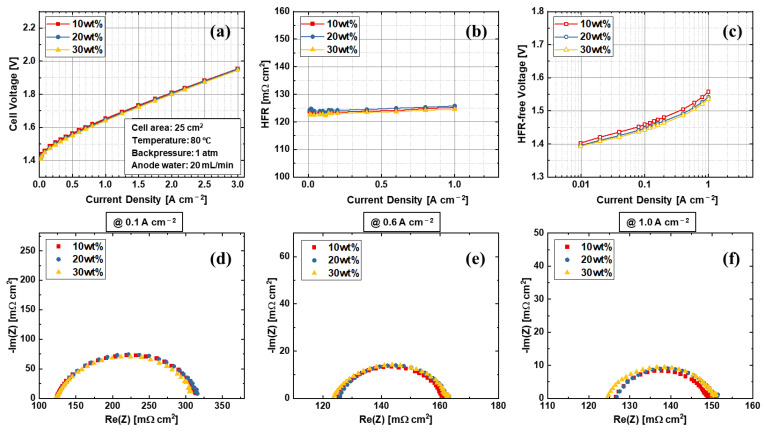
Effects of catalyst weight content in anode electrode ink. (**a**) Performance, (**b**) HFR, (**c**) Tafel slopes, and EIS at (**d**) 0.1 A cm^−2^, (**e**) 0.6 A cm^−2^, and (**f**) 1.0 A cm^−2^.

Figure 10 shows the effects of catalyst weight percentage of the BC + DT electrodes. The results clearly show that the catalyst weight percentage between 10% to 30% has no effects on electrode properties and performances. The HFR of the three samples are identical, as shown in Figure 10b. The electrode made from 10% weight percentage ink shows slightly higher HFR-free voltages, as shown in Figure 10c, but it has nearly no impact on cell performance. When the catalyst weight percentage is low, the ink after the coating needs a longer time to fully dry, thus the ionomer may redistribute during the drying process, which results in different kinetics. However, this effect is so limited that the performances of the electrodes are almost identical. The EIS Nyquist plots (Figure 10d–f) coincide with each other, and no transport loss is found in these electrodes. The results indicate that the PEMWE anode electrode also has a high tolerance of catalyst weight percentage during the fabrication.

## 4. Conclusions

Electrodes are critical components in PEMWE cells which directly determine the performance and efficiency of the PEMWE for hydrogen production. The effects of the electrode fabrication techniques and catalyst ink compositions are investigated in this work. We found that hot press process of the directly ultrasonic sprayed electrode has limited effects on PEMWE cell performance, while the decal transfer process results in slightly worse performance due to higher HFR and kinetics, which can be explained by the thin ionomer skin that formed during the multilayer spray process. For the one path coating electrodes, blade-coated electrode demonstrates a better performance compared to rod-coated electrode, which is mainly due to smaller HFR values, and can be explained by the differences between grooved rod and smooth blade coating tools. The kinetics between blade and rod coating are similar, indicating a good compatibility of the electrode fabrication methods. We have also found that the ink composition has limited impacts on PEMWE performances in a relatively wide range, including ionomer to catalyst ratio (0.1 to 0.3), water to alcohol ratio (1:1 to 3:1), and catalyst weight percentage (10% to 30%). The ink compositions may have impacts on HFR or kinetics, but we have shown that their effects on PEMWE performance are limited, and we have obtained almost identical performance with anode electrodes made from varied ink compositions. This study clearly demonstrates that the PEMWE electrodes have superior good compatibility or redundancy, which can be fabricated by various techniques with very similar performance and kinetics. This high flexibility makes the PEMWE technology a promising approach for green hydrogen production, easier for large-scale manufacturing of electrodes and its commercialization.

## Figures and Tables

**Table 1:** Details of the various anode electrodes in this study.

*Label*	*Substrate*	*Coating Technique*	*Post-Coating Process*	*Ir Loading* *[mg cm^−2^]*	*Pt Loading* *[mg cm^−2^]*
***SP***	Nafion 115	Ultrasonic spray	Null	0.35 ± 0.03	0.2 ± 0.03
***SP + HP***	Nafion 115	Ultrasonic spray	Hot press	0.35 ± 0.03	
***SP + DT***	PTFE	Ultrasonic spray	Decal transfer	0.34 ± 0.04	
***RC + DT***	PTFE	Rod coating	Decal transfer	0.36 ± 0.05	
***BC + DT***	PTFE	Blade coating	Decal transfer	0.35 ± 0.04	

**Table 2:** Results comparison between various electrodes in PEMWE.

	*Cell Voltage@2.0 A cm^−2^* *[V]*	*Avg. HFR* *[mΩ cm^2^]*	*HFR-Free Voltage@0.1 A cm^−2^* *[V]*	*Tafel Slopes* *[mV/dec]*
*SP*	1.80	117 ± 0.6	1.47	58
*SP + HP*	1.79	115 ± 0.6	1.46	57
*SP + DT*	1.81	126 ± 0.7	1.47	59
*RC + DT*	1.82	126 ± 0.8	1.46	60
*BC + DT*	1.79	119 ± 0.7	1.46	56

## Data Availability

Not applicable.
